# DO BLUEBERRIES ACTUALLY IMPROVE COGNITIVE PERFORMANCE? AN ANALYSIS OF PUBLICATION BIAS IN PUBLISHED RESEARCH

**DOI:** 10.1093/geroni/igz038.3126

**Published:** 2019-11-08

**Authors:** Christopher Brydges, Laura Gaeta

**Affiliations:** 1 Colorado State University, Fort Collins, Colorado, United States; 2 California State University, Sacramento, Sacramento, California, United States

## Abstract

A recent published systematic review (Hein et al., 2019) found that consumption of blueberries could improve memory, executive function, and psychomotor function in healthy children and adults, as well as adults with mild cognitive impairment. However, attention to questionable research practices (QRPs; such as selective reporting of results and/or performing analyses on data until statistical significance is achieved) has grown in recent years. The purpose of this study was to examine the results of the studies included in the review for potential publication bias and/or QRPs. p-curve and the test of insufficient variance (TIVA) were conducted on the 22 reported p values to test for evidential value of the published research, and publication bias and QRPs, respectively. The p-curve analyses revealed that the studies did not contain any evidential value for the effect of blueberries on cognitive ability, and the TIVAs suggested that there was evidence of publication bias and/or QRPs in the studies. Although these findings do not indicate that there is no relationship between blueberries and cognitive ability, more high-quality research that is pre-registered and appropriately powered is needed to determine whether a relationship exists at all, and if so, the strength of the evidence to support this association.

